# Antimicrobial Activity of Thermocycled Polymethyl Methacrylate Resin Reinforced with Titanium Dioxide and Copper Oxide Nanoparticles

**DOI:** 10.1155/2021/6690806

**Published:** 2021-01-30

**Authors:** Rashin Giti, Kamiar Zomorodian, Maryam Firouzmandi, Zahra Zareshahrabadi, Sedigheh Rahmannasab

**Affiliations:** ^1^Department of Prosthodontics, Biomaterials Research Center, School of Dentistry, Shiraz University of Medical Sciences, Shiraz, Iran; ^2^Basic Sciences in Infectious Diseases Research Center, Shiraz University of Medical Sciences, Shiraz, Iran; ^3^Department of Operative Dentistry, School of Dentistry, Shiraz University of Medical Sciences, Shiraz, Iran; ^4^Department of Medical Mycology and Parasitology, School of Medicine, Shiraz University of Medical Sciences, Shiraz, Iran; ^5^Student Research Committee, School of Dentistry, Shiraz University of Medical Sciences, Shiraz, Iran

## Abstract

**Aims:**

This study aimed to evaluate the effect of 2.5% and 7.5% copper oxide (CuO) and titanium dioxide (TiO_2_) nanoparticles on the antimicrobial activity of thermocycled polymethyl methacrylate (PMMA) denture base material against standard strains of yeast and bacteria species. *Material and Methods*. In this in vitro study, 150 disk-shaped (10 × 2 mm) specimens of heat-cured PMMA were prepared and divided into five groups (*n* = 30) to be reinforced with 2.5% CuO, 7.5% CuO, 2.5% TiO_2_, or 7.5% TiO_2_ nanoparticles and a control group (without nanoparticle). The specimens were thermocycled, and their antimicrobial activity was assessed against standard strains of yeast including *Candida albicans* and *C. dubliniensis* and oral bacteria species including *Streptococcus mutans, S. sobrinus*, *S. salivarius*, and *S. sanguis*. Data were analyzed with ANOVA and Tukey's post hoc tests (*α* = 0.05).

**Results:**

Both concentrations of CuO and TiO_2_ nanoparticles had significant antimicrobial activity against *S. salivarius, S. sanguis*, and *C. dubliniensis* compared with the control group (*P* < 0.05). Significant differences existed between both 2.5% (*P* = 0.006) and 7.5% CuO (*P* = 0.005) and the control group against *S. mutans*. However, TiO_2_ groups were not significantly different from the control group against *S. mutans.* Concerning *C. albicans*, 7.5% TiO_2_ was the only nanoparticle with significantly higher antimicrobial activity compared with the control group (*P* = 0.043).

**Conclusions:**

Both concentrations of CuO and TiO_2_ were effective antimicrobial agents against *S. salivarius, S. sanguis*, and *C. dubliniensis*, and the concentration of CuO was effective against *S. mutans*. Yet, TiO_2_ was not much effective. Regarding *C. albicans*, only 7.5% TiO_2_ showed efficient antimicrobial activity.

## 1. Introduction

Despite the general decline of edentulism, the increasing elderly population are still facing this problem and increasingly require general dental prosthetic treatments [1]. Polymethyl methacrylate (PMMA) is among the most common structural materials used for the preparation of partial and complete removable dentures, with several desirable properties such as ease of processing, color and chemical stability, durability, cost-effectiveness, and light weight [2]. However, the associating drawbacks include polymerization shrinkage, low mechanical strength, susceptibility to microbial colonization, allergic reactions mainly due to monomer saturation, and degradation of mechanical properties [3].

Adhesion of microorganisms to the denture surface and biofilm formation cause local (e.g., denture stomatitis) and systemic infections (e.g., aspiration pneumonitis) [4, 5]. Surface roughness, porosity, continual denture wearing, and poor denture hygiene are some factors which contribute to the adhesion of microorganisms and biofilm formation on the surfaces of acrylic resins [6]. Despite the improvement in properties and aesthetics of these materials, microorganisms still grow vastly under the acrylic resin bases [7].

Among the most prevalent oral infections, denture-associated oral ulcers (like denture stomatitis) are seen in 73% of prosthodontic patients, most of which are due to *Candida albicans* [6]. Stomatitis can be generally prevented through oral hygiene and denture cleansing, but these simple procedures might be compromised for hospitalized and geriatric patients due to reduced motor dexterity, cognitive impairment, and memory loss. Besides, the common treatment plans, namely, nystatin, fluconazole, and amphotericin B, are short term [8].

There are numerous approaches to enhance the mechanical properties of acrylic appliances and decrease the risk of oral infections and dental caries. Recently, much interest has been directed to inorganic nanoparticles with novel and improved physical, chemical, and biological properties, phenomena, and functionality due to their nanoscale size [9]. Specifically-formulated metal oxide nanoparticles have good antimicrobial activity. Some of these nanoparticles are TiO_2_, SiO_2_, CuO, CeO_2_, Ag, and halloysite nanotube (HNTs), which are incorporated in many biomaterials to induce antimicrobial activity and improve mechanical behavior [10, 11]. The behavior of nanoparticles depends on the size of particles [12].

Studies revealed that PMMA nanocomposite based on functionalized TiO_2_ nanoparticles has better mechanical and antibacterial characteristics, as well as high physicochemical stability, low cost, nontoxicity, and biocompatibility. Moreover, TiO_2_ nanoparticles are catalytic, stable, reliable, and cost-effective and most importantly have antimicrobial effect against *C. albicans*, *Staphylococcus aureus, Pseudomonas aeruginosa*, and *Escherichia coli* [13–15].

Meanwhile, copper nanoparticles have unique physical and chemical properties, besides low preparation cost, which make them widely applicable as heat transfer systems, antimicrobials, ultrastrong materials, sensors, and catalysts [16, 17]. CuO nanoparticles are very active and their high surface-to-volume ratio allows them to easily interact with other particles and increase the antimicrobial efficiency. To use their antibacterial properties, CuO nanoparticles are incorporated in dental materials like resin composites as nanofiller to decline the polymerization shrinkage and bacterial adhesion on the surface and increase the dimensional stability and surface smoothness [16, 18].

Alrahlah et al. [8] reported that TiO_2_ nanoparticles (1, 2, and 3 wt%) improved the antimicrobial behavior of PMMA by reducing the bacterial viability. Copper, gold, titanium, silver and their oxide nanoparticles have antibacterial properties [19]. Amiri et al. [20] detected that nanocopper oxide was highly effective against the examined dental caries bacterial agents and less effective on three species of *Candida*. Hence, CuO nanoparticles were introduced as a candidate control agent for preventing dental caries and dental infections [20]. The study by Argueta-Figueroa et al. [18] showed that Cu nanoparticles had bactericidal effects against *S. aureus, E. coli*, and *S. mutans*. Another study showed that adding TiO_2_ and SiO_2_ nanoparticles (0.5% and 1%) to PMMA imparted antimicrobial activity to the resins, which was more efficient under UVA exposure [14].

The effects of different nanoparticles have been investigated on the physical and antimicrobial properties of PMMA denture base resin. The present study was designed to compare the effect of two concentrations (2.5% and 7.5%) of CuO and TiO_2_ nanoparticles on the antimicrobial activity of PMMA denture base material against standard strains of *Candida* (*albicans* and *dubliniensis*) and *Streptococcus (mutans, sobrinus, salivarius,* and *sanguis)* after thermocycling. The null hypothesis was that adding different concentrations of TiO_2_ and CuO nanoparticles would not influence the antimicrobial activity of thermocycled PMMA denture base resin.

## 2. Materials and Methods

### 2.1. Specimen Preparation

In this in vitro study, 150 heat-cured PMMA specimens (10 × 2 mm) were made with two different concentrations of TiO_2_ and CuO nanoparticles as 2.5% TiO_2_, 2.5% CuO, 7.5% TiO_2_, 7.5% CuO, and control group (without nanoparticles) (*n* = 30 per group). The concentrations of both nanoparticles were selected according to the previous studies [21, 22] and the dimensions of the specimens for the antimicrobial test were based on the ISO standard #1567. To fabricate the PMMA specimens, 30 disk-shaped wax patterns (10 × 2 mm) were invested in dental stone (Fujirock EP; GC, Leuven, Belgium). After the setting of stone, the flasks (61B Two Flask Compress; Handler Manufacturing, Westfield, NJ, USA) containing the wax patterns were opened and dewaxed in boiling water for 5 minutes. TiO_2_ nanoparticles powder (average size = 17 nm, 99.9% purity, Fanavaran Daneshgah, Isfahan, Iran) and CuO nanoparticles (average size = 40 nm, 99.9% purity, Fanavaran Daneshgah, Isfahan, Iran) were weighed by using an electronic balance (Ohaus Corporation, NJ 07058, USA) with accuracy of 0.0001 g, for concentrations of 2.5% and 7.5%. Then, they were mixed with monomer of polymethyl methacrylate (PMMA; SR Triplex Hot, Ivoclar Vivadent, Liechtenstein, Germany) in aseptic conditions. The resulting suspension was stirred by an ultrasonic homogenizer (BioLogics, Inc., Manassas, Virginia 20109, USA) to disperse the nanoparticles in the MMA monomer. Then, the monomer was mixed with PMMA powder in liquid:powder ratio of 1 : 3. Acrylic dough was packed into the mold spaces. After the polymerization, the flasks were cooled in water, opened, and the specimens were removed. Finishing of the specimens was done with silicon carbide discs (Grit 600) by a polishing machine (MetaServ 250 Grinder-Polisher, Buehler, Lake Bluff, IL, USA) at 250 rpm, followed by a cloth wheel and a 0.5 *μ*m diamond suspension. To apply the temperature changes, the acrylic specimens were subjected to 2000 thermal cycles in 5°C and then 55°C water.

### 2.2. Determination of Minimum Inhibitory Concentration (MIC)

Minimum inhibitory concentrations of the TiO_2_ and CuO nanoparticles against the selected microorganisms were determined by using the broth microdilution method according to the guidelines of Clinical and Laboratory Standards Institute (CLSI). To determine the antifungal activities, serial dilutions of TiO_2_ and CuO nanoparticles (128 to 0.25 *μ*l mL^−1^) were prepared in 96-well microtiter plates by using RPMI-1640 media (Sigma, St. Louis, USA) buffered at pH 7.0 with 3-(*N*-morpholino)propane sulfonic acid (Sigma, St. Louis, USA). Stock inoculum suspensions of the yeast and bacteria were prepared by suspending their colonies in 5 mL sterile 0.85% NaCl and the turbidity of the suspensions was adjusted to 0.5 McFarland standard at 530 nm wavelength by the spectrophotometric method to yield stock suspension of 1–5 × 10^6^ cell/mL for yeasts and bacteria. The working suspension of the yeasts and bacteria was prepared by making a 1/1000 and 1/100 dilution of their stock suspension with appropriate broth media, respectively. After adding 0.1 mL of the working inoculums to each well, the plates were incubated in a humid atmosphere at 32°C for 24–48 h and 37°C for 24 h for the yeasts and bacteria, respectively. The wells of the first column of the microtiter plate (containing 200 *µ*L of the uninoculated medium) were considered as a sterility control (blank). Growth controls containing medium with inoculums without the nanoparticle were also used. The lowest concentration of the nanoparticle showing no visible growth was defined as MIC. Each experiment was done in triplicate.

### 2.3. Biofilm Preparation and Growth

**Figure 1:**
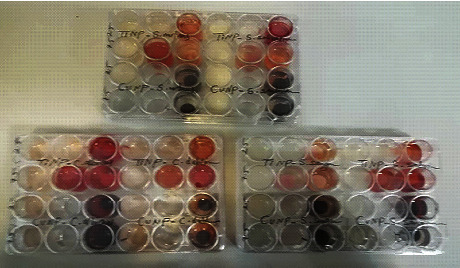
24-cell plates containing acrylic specimens.

Standard strains of microorganism including *C. albicans* (CBS 10261), *C*. *dubliniensis* (777), *S. mutans* (ATCC35668), *S. sobrinus* (ATCC27607), *S. salivarius* (ATCC9222), and *S. sanguis* (ATCC 2908) were cultured on appropriate media. Standard strains of bacteria and yeast were seeded on brain heart infusion agar and Sabouraud dextrose agar (Merck, Germany), respectively, and incubated at 35 ± 2°C between 18 and 24 h. After 24 hours, one loop of yeast and bacteria colonies was transferred to 20 mL Sabouraud dextrose and brain heart infusion broth, respectively, and incubated overnight in an orbital shaker (100 rpm) at 30°C under an aerobic condition. Yeast and bacteria cells were then harvested and washed twice in sterile phosphate-buffered saline (PBS, 0.8% w/v), sodium chloride (0.02% w/v, Merck, Germany), KH_2_PO_4_ (0.31% w/v, Merck, Germany), Na_2_HPO_4_.12H_2_O (0.02% w/v, Merck, Germany), and KCl (Panreac, Madrid, Spain) with pH 7.4. Then, the cell densities were adjusted at 0.5 McFarland standards by a spectrophotometric method. In order to determine the inhibitory effect on the biofilm formation of *Candida* and oral *Streptococcus*, 24-cell culture microtiter plates were used. For *Candida* species, 1 mL of RPMI-1640 was added into the first and 0.5 mL to the other columns. Then, resin disks containing different concentrations of nanoparticles (sterilized with UV rays) were placed into the appropriate wells. To assess the potential of the resin disks with nanoparticles on the mentioned bacteria strains biofilm formation, an overnight culture of each was grown in tryptic soy broth (TSB, Merck, Germany) for 18 to 20 hours at 37°C. The suspensions were adjusted with TSB to 0.5 McFarland as measured by absorbance (0.08 to 0.1 at 625 nm) in a spectrophotometer corresponding to approximately 10^8^ CFU mL^−1^. Exposure of *Candida* and nanoparticles was performed as done for bacteria. Then, the plates were placed at 30°C for 48 h to form biofilm (Figure 1). After 48 hours, the plates were moved out of the incubator; the contents of each well were expelled and washed twice with sterile phosphate buffered saline.

### 2.4. Assessing Biofilm Formation

Biofilm formation was assayed by using a 2,3-bis(2-methoxy-4-nitro-5-sulfo-phenyl)-2H-tetrazolium-5-carbox-anilide (XTT, Sigma, USA) reduction assay. The XTT was prepared as a saturated solution at a concentration of 0.5 mg mL^−1^ in Ringer's lactate. The solution was filter-sterilized through a 0.22 *µ*m pore size filter, divided into aliquots, and then stored at −70°C. Prior to each assay, an aliquot of the XTT stock solution was thawed and treated with menadione sodium bisulfite (10 mmol L^−1^ prepared in distilled water; Sigma, USA) to obtain a final concentration of 1 µmol L^−1^ of menadione. A 500 *µ*L aliquot of XTT-menadione was then added to each prewashed biofilm and wells that contained disks (5 disks for each microorganism) treated with CuO and TiO_2_ nanoparticles and incubated for 4 h at 35°C in dark to measure background XTT levels. After adding 500 *μ*L of the prepared compound of XTT-menadione, the content of the wells was transferred to another plate and their spectral absorbance at a wavelength of 570 nm was evaluated by a multiwell scanning spectrophotometer (POLARstar Omega, Germany). All trials were performed in triplicate to maximally reduce the error rate [23]. Resin disks without nanoparticles in wells containing media and organism were used as positive control, while resin disks in wells with media served as the negative controls. Inhibition of biofilm formation was calculated as follows:

(1) $$\begin{array}{c} \;\frac{\text{optical density of the growth control}-\text{optical density of the sample}}{\text{optical density of the growth control}}\times 100. \end{array}$$

### 2.5. Data Analysis

Data were analyzed by using SPSS software (v.24, IBM product, Chicago, IL, USA). Numerical data were presented as means and standard deviations, explored for normality by using Kolmogorov–Smirnov test. ANOVA and Tukey's post hoc tests were used as appropriate (*α* = 0.05).

## 3. Results

Nanoparticles of CuO and TiO_2_ successfully inhibited the growth of the tested standard strains of yeast and bacteria at concentrations ranging from 32 to 64 *µ*g mL^−1^. Means and standard deviations of optical density and biofilm inhibition of the study groups for each strain of *Streptococcus* and *Candida* are presented in Tables 1 and 2. The higher optical density and lower percentage of biofilm inhibition shows lower antimicrobial activity against specific strain.

**Figure 2:**
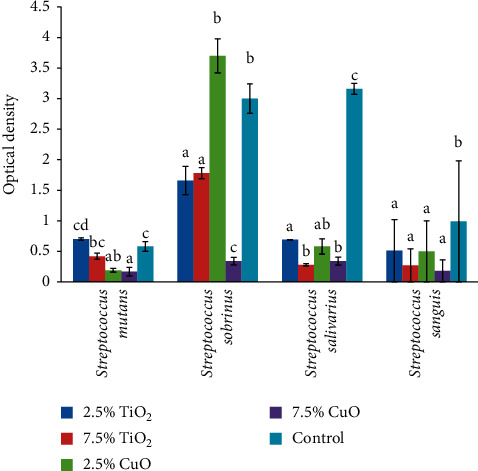
Means and standard deviations (*n* = 5) of optical density of the study groups against different strains of *Streptococcus*.

The result of one-way ANOVA revealed significantly different optical density values (antimicrobial activity) among the groups against all strains of *Streptococcus (mutans* (*P* < 0.001), *sobrinus* (*P* < 0.001), *salivarius* (*P* < 0.001), and *sanguis* (*P* = 0.002)) and *Candida* (*albicans* (*P* = 0.045) and *dubliniensis* (*P* < 0.001)). Tukey's post hoc tests showed that both concentrations of CuO had significantly higher antimicrobial activity against *S. mutans* than the control and TiO_2_ nanoparticle groups. However, the two concentrations of CuO nanoparticles were not significantly different (*P* = 0.986). Nor was any significant difference between 2.5% TiO_2_ (*P* = 0.316) and 7.5% TiO_2_ (*P* = 0.173) and the control group (Figure 2).

**Figure 3:**
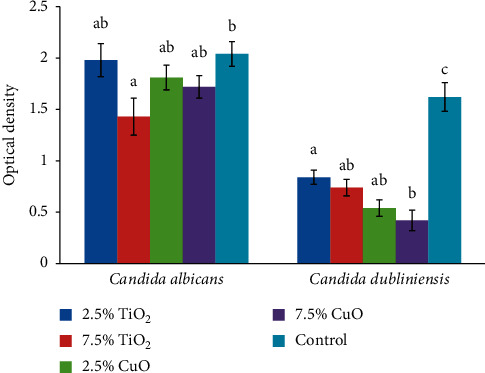
Means and standard deviations (*n* = 5) of optical density of the study groups against different strains of *Candida*.

Concerning the *S. sobrinus*, 2.5% TiO_2_ had significantly higher antimicrobial effect than both the control (*P* = 0.006) and 2.5% CuO group (*P* = 0.001). Increasing the concentration of CuO from 2.5% to 7.5% significantly improved its antimicrobial effect (*P* < 0.001). Regarding the *S. salivarius*, both concentrations of both nanoparticles had significantly higher antimicrobial activity compared with the control group (2.5% TiO_2_ (*P* < 0.001), 7.5% TiO_2_ (*P* < 0.001), 2.5% CuO (*P* < 0.001), and 7.5% CuO (*P* < 0.001)). Considering the *S. sanguis*, all groups had significantly higher antimicrobial activity than the control group (2.5% TiO_2_ (*P* = 0.021), 7.5% TiO_2_ (*P* = 0.004), 2.5% CuO (*P* < 0.020), and 7.5% CuO (*P* < 0.02), with no significant difference among the nanoparticles (*P* > 0.05). Relating to *C. albicans*, only 7.5% TiO_2_ had significantly higher antimicrobial activity than the control group (*P* = 0.043). For *C. dubliniensis*, both concentrations of both nanoparticles had significantly higher antimicrobial activity than the control group (2.5% TiO_2_ (*P* = 0.003), 7.5% TiO_2_ (*P* = 0.002), 2.5% CuO (*P* = 0.001), and 7.5% CuO (*P* < 0.001)) with no significant difference between the two concentrations of nanoparticles (*P* > 0.05) (Figure 3).

## 4. Discussion

The null hypothesis was rejected as adding different concentrations of TiO_2_ and CuO nanoparticles affected the antimicrobial properties of PMMA denture base resin after thermocycling. Many factors affect the adhesion of microorganisms and biofilm formation on the surfaces of acrylic resins, such as surface roughness, porosity, continual denture wearing, and poor denture hygiene [6]. This material is susceptible to colonization by various microbial species including *C. albicans*, *C. glabrata*, and Gram-positive/negative organisms [13], necessitating the development of a denture with self-antimicrobial features. Oral temperature changes might be affected by routine activities like eating, drinking, breathing, and heat pressures that cause aging in denture and alter its properties. Like no other previous study, the present one considered these temperature changes to the acrylic specimens through thermocycling.

### 4.1. PMMA + CuO

The present results showed that both concentrations of CuO nanoparticles had significant antimicrobial activity against almost all strains of *Streptococcus* and *C. dubliniensis*. Similarly, Argueta-Figueroa et al. [18] confirmed the bactericidal effect of Cu nanoparticle against *S. aureus, E. coli*, and *S. mutans*. Ahamed et al. [24] detected that CuO nanoparticles could be externally used as antibacterial agents in surface coatings on various substrates to prevent microorganisms from attaching, colonizing, spreading, and forming biofilms in indwelling medical devices. It also had excellent antimicrobial activity against various bacterial strains (*Escherichia coli, Pseudomonas aeruginosa, Klebsiella pneumonia*, and *Staphylococcus aureus*). Benoit et al. [25] reported CuO nanoparticles as proper alternatives to control biofilm formation within the oral cavity.

In this study, neither concentration of CuO nanoparticles affected the antimicrobial activity against *C. albicans*. This was in agreement with the study conducted by Amiri et al. [20], which showed that nanocopper oxide had high antimicrobial effect against the examined dental caries bacterial agents and lower effect on the three species of *Candida*. Another study showed that the antimicrobial action of CuO nanoparticles on all the three species of *C. albicans*, *C. krusei*, and *C. glabrata* was not as effective as that on cariogenic bacteria; yet they might be good candidate control agents for preventing cariogenic activity and other dental infections [26]. The antimicrobial activity of CuO nanoparticles is due to their close interaction with microbial membranes and the release of metal ions. The nanoparticles are slowly oxidized, release copper ions, and produce toxic hydroxyl free radicals when close to the lipid membrane. Free radicals separate the lipids from the cell membrane through oxidation and destroy the membrane [27].

### 4.2. PMMA + TiO_2_

This study found that neither concentration of TiO_2_ had significant effect against *S. mutans*. Moreover, both concentrations of TiO_2_ nanoparticles had significant antimicrobial activity against *S. salivarius, S. sanguis*, and *C. dubliniensis*. Concerning the *C. albicans*, 7.5% TiO_2_ was the only nanoparticle with significant antimicrobial effect. This was consistent with Abdulrazzaq Naji et al.'s study [13], which suggested PMMA/TiO_2_ nanotube composite as a promising material for antimicrobial approaches. Another study showed that adding 3 wt% TiO_2_ nanoparticles to PMMA produced a positive antimicrobial effect, reduced the microbial number, which prevented quorum sensing, and thereby halted plaque formation on PMMA/TiO_2_ nanocomposite surface [28]. Likewise, Gad and Abualsaud [29] showed that all concentrations of TiO_2_ nanoparticles (0.4%, 1%, and 2.5%) inhibited the growth of *Candida*.

TiO_2_ nanoparticles have an intrinsic antimicrobial property due to the production of cytotoxic oxygen radicals. When exposed to UV-light in the presence of oxygen and water, these particles decompose and oxidize other organic and inorganic compounds [11]. Therefore, they can be considered as antimicrobial additive. The integration of TiO_2_ nanoparticles to PMMA reduces the porosity of the denture bases. Besides, TiO_2_ nanoparticles inhibit the adherence of cariogenic bacteria in planktonic phase and further phases of biofilm formation [30]. Hence, increasing the TiO_2_ ratio in PMMA-TiO_2_ improves the antimicrobial activity of PMMA by drastically decreasing bacterial adherence [29].

Alrahlah et al. [8] found that adding only 3% TiO_2_ nanoparticles decreased the bacterial cell attachment of *E. faecalis* and *P. aeruginosa* by 50 to 90%. A different study reported that coating the implants with TiO_2_ nanoparticles inhibited the adhesion and growth of bacteria such as *S. mutans*, *S. epidermis*, and *E. coli*, besides preventing the inflammation around the implants [14]. A significant reduction was also noted in bacterial adhesion and colonization of *S. mutans*, *S. salivarius*, and *S. sanguis* on anatase-coated titanium of dental implant abutments [31].

Increasing the concentration of TiO_2_ from 2.5% to 7.5% did not significantly increase the antimicrobial effect against *S. sobrinus*, *S. sanguis*, *C. albicans*, and *C. dubliniensis*. It was in line with Totu et al.'s study [32], which found that incorporating even small amounts of 0.4% TiO_2_ nanoparticles to a 3D-printed PMMA denture prevented the colonization of microorganisms and further biofilm formation. Another study showed that adding TiO_2_ and SiO_2_ nanoparticles (0.5% and 1%) to PMMA imparted antimicrobial activity to the resins, being even more efficient under UVA exposure [33].

According to the present findings, the clinicians and technicians are suggested to add either 2.5% or 7.5% of CuO in combination with 7.5% TiO_2_ nanoparticle to heat-cured PMMA denture bases and improve the antimicrobial activity of dentures against almost all species of the *Candida* and *Streptococcus*, which are the most common microorganisms colonized on denture base materials.

The limitation of this study was assessing only two concentrations of the two types of nanoparticles. Moreover, the antimicrobial effect of these nanoparticles was not evaluated in the clinical situation in presence of saliva. Further studies are recommended to evaluate the antimicrobial properties of other concentrations and types of nanoparticles and the combination of these nanoparticles against different oral microorganism in the clinical situations.

## 5. Conclusions

Considering the findings and limitations of the present study, the following can be concluded:

1. Adding both 2.5% and 7.5% concentrations of CuO and TiO_2_ nanoparticles to heat-cured PMMA denture base material can obviously increase the antimicrobial activity against *S. salivarius, S. sanguis*, and *C. dubliniensis*
2. Both concentrations of CuO nanoparticles can significantly improve the antimicrobial activity of PMMA against *S. mutans*; however, TiO_2_ nanoparticles do not alter the antimicrobial activity of PMMA against *S. mutans*
3. Concerning *C. albicans*, 7.5% TiO_2_ is the only nanoparticle that can increase the antimicrobial activity of PMMA against this microorganism
4. Adding 2.5% or 7.5% concentrations of CuO nanoparticles in combination with 7.5% TiO_2_ to heat-cured PMMA denture base materials can effectively inhibit the growth of different species of *Streptococcus* and *Candida*

## Figures and Tables

**Table 1:** Means ± standard deviations, biofilm inhibition (%), and multiple comparisons of optical density of the study groups for bacteria species.

Groups	Factors	Bacteria species			
		*S. mutans*	*S. sobrinus*	*S. salivarius*	*S. sanguis*
2.5% TiO_2_	Optical density	0.70 ± 0.02^cd^	1.66 ± 0.23^a^	0.69 ± 0.00^a^	0.51 ± 0.07^a^
	Biofilm inhibition	25%	48%	80%	70%

7.5% TiO_2_	Optical density	0.42 ± 0.06^bc^	1.78 ± 0.09^a^	0.28 ± 0.01^b^	0.27 ± 0.07^a^
	Biofilm inhibition	18%	43.7%	93%	79%

2.5% CuO	Optical density	0.19 ± 0.03^ab^	3.70 ± 0.21^b^	0.58 ± 0.12^ab^	0.50 ± 0.13^a^
	Biofilm inhibition	60%	6%	83%	44%

7.5% CuO	Optical density	0.17 ± 0.07^a^	0.34 ± 0.06^c^	0.34 ± 0.06^b^	0.18 ± 0.01^a^
	Biofilm inhibition	66%	89%	90%	80%

Control	Optical density	0.58 ± 0.08^c^	3.00 ± 0.24^b^	3.16 ± 0.09^c^	0.99 ± 0.08^b^

Vertically within groups, different superscript lowercase letters indicate significant differences between groups in the same column (*P* ≤ 0.05).

**Table 2:** Means ± standard deviations, biofilm inhibition (%), and multiple comparisons of optical density of the study groups for *Candida* species.

Groups	Factors	*Candida* species	
		*C. albicans*	*C. dubliniensis*
2.5% TiO_2_	Optical density	1.98 ± 0.16^a,b^	0.84 ± 0.07^a^
	Biofilm inhibition	3 %	46%

7.5% TiO_2_	Optical density	1.43 ± 0.18^a^	0.74 ± 0.08^a,b^
	Biofilm inhibition	31%	56%

2.5% CuO	Optical density	1.81 ± 0.12^a,b^	0.54 ± 0.08^a,b^
	Biofilm inhibition	13%	66%

7.5% CuO	Optical density	1.72 ± 0.11^a,b^	0.42 ± 0.10^b^
	Biofilm inhibition	18%	73%

Control	Optical density	2.04 ± 0.12^b^	1.62 ± 0.14^c^

Vertically within groups, different superscript lowercase letters indicate significant differences between groups in the same column (*P* ≤ 0.05).

## Data Availability

All the data used to support the findings of this study are available from the corresponding author upon request.
